# The Radcliffe Infirmary, Oxford

**Published:** 1893-03-04

**Authors:** 


### THE RADCLIFFE INFIRMARY, OXFORD.

The Sheldonian Theatre at Oxford was filled to overflowing on Wednesday, the 1st inst., on the occasion of a meeting held in aid of the building fund of the Radcliffe Infirmary. Lord Salisbury, the Chancellor of the University, presided, and was greeted on his entrance with ringing cheers from the large audience, representative of the county, city, and University, assembled. Among those present were the Vice-Chancellor, the Warden of New College, the Warden of All Souls, the Principal of St. Mary Hall, the Principal of Brazenose, the Principal of Manchester New College, the Provost of Oriel, the Provost of Queen's, the Provost of Worcester, the Master of Pembroke, the Master of University, the President of Trinity, the President of Corpus, the President of Magdalen, the Rector of Exeter, the Senior and Junior Proctors, Archdeacon Palmer, Canon Bright, Sir Henry Acland, Lord Saye and Sele, Professors Max Muller, Legge, Burrowes, Dicey, and Nettleship, Mr. G. H. Morrell, the Mayor of Oxford (Mr. Lucas), the Sheriff (Mr. Taphouse), and several of the Aldermen.

Lord Salisbury, who was greeted with loud and prolonged cheers, in the course of an eloquent address, said there was a most candid quotation in the circular which had been sent round, from which he would like to read an extract. It was an extract from a friendly critic called "The Hospital Annual," and it showed how severe the vigilance was which scientific men exercised over each other. (Laughter.) The language in which this book, which had a very large circulation, described the infirmary was this: "Notwithstanding this admission, this wealthy county, this illustrious University has remained in a state of quiescence and seems content to allow its hospital to remain inefficient, inadequate, and almost a bye-word amongst the hospitals of this country at the present time. This is an age in which humbug is recognised and nailed to the post." He could not concur in the latter observation—(laughter)—but if it was desired that the hospital should no longer remain inefficient, inadequate, and a bye-word, he was sure that the people of Oxford would come forward on this occasion in order to assist those who desired to place it in its proper position. Its proper position in that city of learning was to be a model to all other hospitals in the country. Its proper position was to be equipped with all that science and all that practice could furnish to make it the most efficacious of the means for bringing medical knowledge to bear on the relief of the sufferings of mankind. This was the aim at which the trustees should point. He felt, however, that something more than the aim of promoting a philanthropic end was in the minds of those who set that movement on foot. He could not help connecting that movement with a desire on their part to strengthen that hold which the older sciences—the sciences which relied most upon observation—had recently exercised upon the intellect of men, and the homage which they had exacted from that University. He believed that if they responded munificently to the appeal which was made to them they would do something more than place that infirmary in a position in which it would not be ashamed of rivalling the county hospitals in its neighbourhood. They would be taking a long step towards introducing the closer cultivation of one of the greatest of sciences—the science of medicine—in that ancient University. (Applause.) Of all the sciences medicine was the only one which was but another name for a work of mercy—there was no other that was so closely linked with the relief of human suffering and the remedying of human calamity in its worst and most overwhelming form, and for these reasons it would be a matter of great satisfaction if the Radcliffe Infirmary should be increased; should be thoroughly equipped; should spread and grow; should make a powerful school of medicine, and if that school of medicine should exercise a great influence upon the tone, temper and direction of the studies of the University. (Applause.) In conclusion, his Lordship said that in commending the appeal on behalf of the infirmary to their consideration, he was doing more than what he would be singularly unfit for; he was doing more than preaching a charity sermon to them; he was asking them to help that which contained a most brilliant promise for the intellectual future of science, and of a University by which science ought to be cultivated, and where science ought to reign. He was asking them to help forward those habits of research, those sober, argumentative, dialectical methods of thought; those earnest efforts for the benefit of all mankind, high and low, rich and poor, which had adorned the memory, and made practicable the work of those who had gone before them in that place which entombed so many great reputations. (Applause.) It might be that he was indulging in an Alcanaza's dream, but at all events it was attractive enough to have induced him to draw their attention to that side of the appeal. In helping forward the infirmary he entreated his hearers to remember that they were not only subscribing to that which was a most useful and valuable institution, they were not only removing that which was a disgrace to the County and University of Oxford, but they were adding another row of bricks to that mighty edifice of intellectual supremacy and culture which had made Oxford famous before all other places in the world. (Loud and prolonged applause.)

On the motion of Professor Dicey, seconded by Lord Dillon, it was unanimously resolved:—

"That, in the opinion of this meeting, the Radcliffe Infirmary being the chief hospital for Oxford and the large surrounding district, the county, city, and University should combine to maintain its efficiency, and to raise it to the standard required by recent advances in hospital management."

The Master of University, the chairman of the Committee of Management, proposed the following resolution:—

"That this meeting approves generally of the scheme of the committee, consisting of the removal of the sick from the old building to well-appointed modern wards, and the renovation of the old building, and its conversion into a suitable administrative building."

He said that all present were more or less acquainted with the outside of the Radcliffe Infirmary. It was a very venerable building—he could hardly call it a beautiful building; but it was a strongly-built building. It was 130 years of age, and the very fact of saying that they were trying to carry on a hospital in a building which was designed by people 130 years ago was equivalent to saying that they were trying to do with very second-rate apparatus. It must, however, be borne in mind that many years ago his predecessors on the Committee of Management had seen that the building was not a convenient one, that the upstairs garrets were but garrets and not fit for wards, and they had entered upon a course of improvement. Accordingly they carried out a considerable number of improvements, but, as not infrequently happened when one set to work to patch up a very old building, these improvements in some instances certainly had not produced the very desirable result which was wanted, and especially so, as the work was carried on piecemeal. About eleven years ago, however, a fixed and definite plan was arrived at by which a corridor was to be built on the strip of land at the back of the hospital running east and west, with wards branching off it. That scheme had been carried out as far as and when funds had allowed, that was to say, an excellent pair of children's wards had been erected by Mr. Waddilove and Mrs.

Coombes, and a female ward built out of funds received in Jubilee year. All that the hospital now wanted was the men's wards. It might be asked, why was it necessary to erect such wards if there were still wards in the old house? The answer was because the old house had been getting worse and worse in the passage of years. If unfit for patients thirty years ago it was hardly fit for patients now. Inconvenient and bad in many respects for wards as it was, the old building, however, was admirably suited for being turned into an administration block, but it required alteration. The nurses' and servants' rooms were bad, and the means of storage were indifferent; but all this could be put right if the building was renovated.

Sir Henry Acland, in seconding, said that the County Hospital should be the place where everything was perfect in its kind, from the work in the kitchen to that in the surgery.

The resolution having been carried unanimously,

Lord Salisbury announced that the subscriptions to the building fund amounted to £4,169.

A vote of thanks to Lord Salisbury terminated the proceedings.

